# Lightweight Potential of Anisotropic Plate Lattice Metamaterials

**DOI:** 10.3390/ma17102354

**Published:** 2024-05-15

**Authors:** Martin Maier, Christoph Stangl, Holger Saage, Otto Huber

**Affiliations:** Competence Center for Lightweight Design (LLK), University of Applied Sciences Landshut, 84036 Landshut, Germany; martin.maier@haw-landshut.de (M.M.); christoph.stangl@haw-landshut.de (C.S.); holger.saage@haw-landshut.de (H.S.)

**Keywords:** anisotropic plate lattice metamaterials, lightweight potential, buckling, wall thickness optimization, stress concentrations, production-oriented cell architecture

## Abstract

Additive manufacturing enables the production of lattice structures, which have been proven to be a superior class of lightweight mechanical metamaterials whose specific stiffness can reach the theoretical limit of the upper Hashin–Shtrikman bound for isotropic cellular materials. To achieve isotropy, complex structures are required, which can be challenging in powder bed additive manufacturing, especially with regard to subsequent powder removal. The present study focuses on the Finite Element Method simulation of 2.5D anisotropic plate lattice metamaterials and the investigation of their lightweight potential. The intentional use of anisotropic structures allows the production of a cell architecture that is easily manufacturable via Laser Powder Bed Fusion (LPBF) while also enabling straightforward optimization for specific load cases. The work demonstrates that the considered anisotropic plate lattices exhibit high weight-specific stiffnesses, superior to those of honeycomb structures, and, simultaneously, a good de-powdering capability. A significant increase in stiffness and the associated surpassing of the upper Hashin–Shtrikman bound due to anisotropy is achievable by optimizing wall thicknesses depending on specific load cases. A stability analysis reveals that, in all lattice structures, plastic deformation is initiated before linear buckling occurs. An analysis of stress concentrations indicates that the introduction of radii at the plate intersections reduces stress peaks and simultaneously increases the weight-specific stiffnesses and thus the lightweight potential. Exemplary samples illustrate the feasibility of manufacturing the analyzed metamaterials within the LPBF process.

## 1. Introduction

The advent and evolution of additive manufacturing methods have enabled the production of intricate structures that would be unattainable through conventional manufacturing processes [1]. A widely employed additive manufacturing technique for metals is the Laser Powder Bed Fusion (LPBF) process. In the course of the LPBF process, a powdered metallic feedstock is incrementally deposited, and laser energy is applied to selectively melt specific regions of the powder bed, thereby fusing individual powder particles. The LPBF process has been extensively investigated in studies such as [2,3,4,5]. The influences of the manufacturing process on the quality of the end product are still a subject of research [6,7,8,9]. According to [2], various base materials, including aluminum alloys, nickel-based alloys, titanium alloys, and stainless steel alloys, are suitable for the utilized powder. The present work employs the aluminum alloy AlSi10Mg, a material extensively discussed in the literature [5,7,10,11,12]. In ref. [13], the rationale behind the use of an aluminum alloy is elucidated. An essential consideration for lightweight construction is the low density of aluminum, coupled with its concurrent high stiffness and strength.

Using the LPBF process, weight- and stiffness-optimized geometries inspired by complex structures found in nature are developed, as demonstrated in studies such as [14,15,16]. Following a similar approach, studies such as [17,18] pursued force-flow-optimized structural development by replacing a solid component with a complex lattice of struts arranged according to the natural flow of forces. The manufacturing of cellular structures consisting of many repeating, uniform cells is also feasible. Such lattice metamaterials, widely discussed in publications [6,8,10,19,20], represent an area of significant research interest. Various forms of lattice unit cells are extensively researched, and they can be constructed from struts [21,22,23,24], plates [19,25,26,27], or curved surfaces [28,29,30,31,32]. Of central interest is the determination of the homogenized elastic material properties of lattice structures. This is typically accomplished numerically using the Finite Element Method (FEM) and experimentally [19,28,31].

The numerical homogenization of a lattice metamaterial is typically performed using the Finite Element Method (FEM) and periodic boundary conditions (PBCs). This particular form of boundary conditions depicts a representative volume element (RVE) as if it were embedded in an infinitely extending metamaterial. In the case of a lattice structure, such an RVE is composed of one or more unit cells. In refs. [33,34,35], it is demonstrated that homogenization through PBCs is the most accurate and efficient approach to the numerical homogenization of multi-phase materials using FEM. Comprehensive investigations into the theoretical foundations and numerical implementation of PBCs were performed by S. Li et al. in [36,37,38,39,40].

From [25,31], it is evident that plate lattice metamaterials are superior to strut structures in terms of weight-specific elastic stiffness and also exhibit a significantly higher energy absorption rate. In ref. [25], it is demonstrated that isotropic plate lattice metamaterials can achieve the upper Hashin–Shtrikman bound [41], representing a boundary for the maximum achievable elastic material properties of an isotropic cellular material. To achieve isotropy in a lattice structure, as demonstrated in [19,25], highly complex cell geometries with numerous interlocking plates are required. This significantly complicates the removal of powder residues resulting from the LPBF process, as the complex cell geometry leads to the formation of excessively enclosed void spaces. To enable de-powdering, additional small holes need to be introduced, which cause stress concentration problems. In response to this challenge, ref. [27] proposes the development of plate lattice metamaterials with an already semi-open cell topology. In addition to isotropic materials, there is a wide range of anisotropic materials that are being intensively researched to take advantage of their directional properties. These include, for example, porous materials [42] and biological tissues in medical research [43,44]. In ref. [24], anisotropic strut lattice structures are considered. In ref. [19,27], anisotropic plate lattice metamaterials are investigated, and it is shown that they can be transformed into isotropic metamaterials through the adjustment of certain geometric parameters.

Due to the characteristic structure and thin-walled nature of lightweight lattice structures, further research is required regarding their stability behavior. Numerical and experimental investigations on this matter were conducted in studies such as [21,45,46] and carried out in [21,46] using nonlinear Finite Element Method (FEM) analysis. The FEM simulations performed in [21,45,46] depict a finite lattice structure that is compressed between two supports, simulating a compressive test scenario without using a RVE with PBCs.

The objective of the present study is to numerically investigate and optimize the stiffness and strength of plate lattice metamaterials by deliberately incorporating structural anisotropy for specific multiaxial load cases and to prove their manufacturability via the LPBF process. The focus is placed on 2.5D semi-open cell geometries with a simple structure to ensure easy removal of the powder resulting from the LPBF process. In this context, plate lattice metamaterials with an orthotropic material behavior and a 90° axisymmetry about a distinguished axial direction are considered. Such a metamaterial exhibits identical effective elastic stiffness properties in the two transversally normal directions forming a Cartesian coordinate system with the axial direction. This enables a more versatile range of applications in the case of a multiaxial loading. The widely studied and continually investigated honeycomb structures [47,48,49] also exhibit a similar structural behavior if they possess a regular hexagonal configuration [50]. Therefore, the weight-specific stiffnesses should be analyzed and compared with those of honeycomb structures and, additionally, with those of other known plate lattice metamaterials.

Furthermore, this study explores the possibilities of performing a stability analysis based on a unit-cell model with periodic boundary conditions. Another main objective of this work is to conduct a precise examination of the stress concentrations occurring in such lattice structures and investigate how they can be reduced. To achieve this, the resulting stress concentrations and weight-specific strengths of the lattice structures are compared.

## 2. Materials

### 2.1. Constituent Material

The three lattice metamaterials under consideration are intended to be made of AlSi10Mg. The isotropic elastic modulus *E* and the yield strength $\sigma_{Y}$ of AlSi10Mg are based on the material data published by Aconity3D [51] for the as-built state. For Poisson’s ratio ν, values between 0.31 and 0.39 were determined in [11,52] based on experiments conducted on additively manufactured specimens of AlSi10Mg. For the subsequent considerations, a value of 0.34 is employed. All material data used in this work are summarized in Table 1.

### 2.2. The Architecture and Manufacturing of the Anisotropic Plate Lattices

**Figure 1:**
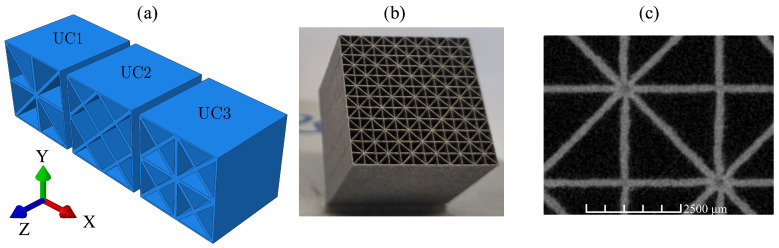
(**a**) The architectures of the three considered plate lattice cells, UC1, UC2, and UC3, (**b**) an exemplary specimen measuring 30 mm × 30 mm × 30 mm, composed of unit cells of type UC1 fabricated using the LPBF process, and (**c**) CT scan of the specimen.

The structures of the three considered lattice unit cells, UC1, UC2, and UC3, are illustrated in Figure 1a. These cells were selected due to their high lightweight potential for the basic load cases of uniaxial tension/compression and pure shear, as well as their favorable manufacturability using the LPBF process with aluminum powder. It is evident that the three cells exhibit a cube-based foundational structure and are symmetric with respect to all three coordinate planes and 90° axisymmetric about the axial direction z. The dimensions of the unit cells are 5 mm × 5 mm × 5 mm, resulting in a volume of the entire unit cell of $V_{UC}=$ 125 mm^3^. This size enables its use in typical mechanical engineering applications in a way that enough unit cells are present to treat it as an effectively homogeneous metamaterial. The actual solid material volume $V_{S}$ is dependent on the chosen wall thickness. Wall thicknesses of approximately t= 0.2 mm are targeted because of the high lightweight potential and, as tests have shown, good producibility using the LPBF process with aluminum powder. The relative density

(1) $$\rho^{*}=\frac{V_{S}}{V_{UC}}$$

is used to characterize the weight of the unit cells. The outer plates of the unit cells shown in Figure 1a, in comparison to the inner ones, exhibit only a thickness of $\frac{t}{2}$. This is due to the fact that a unit cell represents a periodically repeating structure of a larger domain. This implies that a geometrically identical unit cell is adjacent on each side. Consequently, after the attachment of another unit cell, the outer plates also exhibit the full wall thickness.

The three unit cells each exhibit a deliberately chosen arrangement of plates, ensuring that each cell is particularly well suited for specific loading conditions. This is achieved by orienting the plates in the direction of the principal stresses. The z-axis represents the axial direction for all cells. In cell UC1, three plates are oriented in the xz-plane and three plates are oriented in the yz-plane because the x- and y-directions represent the principal stress directions under tension or compression along these axes. The additional diagonal plates are included to provide some stiffness against inevitable variations in the direction of loading. In contrast, for cell UC2, most of the plates are oriented at 45° to the x- and y-axes, corresponding to the principal stress direction for shear loading in the xy-plane. The surrounding plates oriented in the xz- and yz-planes in cell UC2 allow for a superposition of additional tensile/compressive loads in the transverse directions. Cell UC3 represents a superposition of cells UC1 and UC2 and is suitable for tensile/compressive loading in the x-, y-, and z-directions, as well as for shear loading in the xy-, xz-, and yz-planes. From Figure 1a, it is evident that unit cell UC3 is composed of four repeating smaller structures. It would thus be possible to consider UC3 using an even further reduced model as an RVE. As Section 4.2 will demonstrate, forgoing further reduction proves advantageous in the stability analysis.

Figure 1b shows a specimen of size 30 mm × 30 mm × 30 mm consisting of cells of type UC1 with a target wall thickness of 0.2 mm as a demonstration of manufacturability using the LPBF process. The specimen was manufactured using the Aconity Midi LPBF machine [53]. During the fabrication process, only straight lines were exposed with the laser to construct the walls, and the wall thickness was controlled through process parameters. For the desired wall thickness of 0.2 mm, a layer thickness of 30 μm, a laser power of 500 W, a scanning speed of 2200 mm $s^{-1}$, and a spot diameter of 0.160 mm were employed. The build platform was maintained at a constant temperature of 200 °C, and the process was conducted under an argon atmosphere. Figure 1c presents a CT scan of the specimen. The statistical evaluation of 100 individual measurements of the wall thickness resulted in an average wall thickness of 0.204 mm.

The open structure in the z-direction allows for easy removal of powder residues, which inevitably result from the LPBF process. This also results in a wide range of possible applications for the cells. For example, a sandwich structure with the considered cells as the core and the z-axis of the cells parallel to the top and bottom layers can be additively manufactured in one step via integral construction as a single firmly bonded component. This eliminates the need for subsequent bonding of the top and bottom layers and thus also eliminates a potential strength problem. Furthermore, due to the open structure in one direction, applications in the field of fluid mechanics, heat transfer, acoustics, or vibration technology are possible.

Due to the 2.5D structure of the cells, manufacturing through extrusion would theoretically be possible. However, the LPBF process allows for finer geometries and, as experiments have shown, even wall thicknesses below 0.2 mm are achievable, whereas aluminum extrusion typically yields only larger wall thicknesses [54,55,56,57,58]. Furthermore, the LPBF process enables the addition of plate elements perpendicular to the axial direction. This allows, for example, localized stiffening to counteract buckling or permits the targeted manipulation of flow in the case of fluid mechanical applications. In this way, it is possible to significantly improve the performance of a heat exchanger by increasing the convection surface and by increasing the heat transfer coefficient via turbulent flow by introducing transversal ribs as turbulence generators.

## 3. Computational Methods

### 3.1. The Numerical Homogenization of the Linear Elastic Properties of the Plate Lattice Structures

Due to their planes of symmetry with respect to the three coordinate planes, the three lattice cells exhibit an orthotropic elastic material behavior on the macroscopic level. The stress–strain relationship can be expressed as

(2) $$\left[\sigma^{0}\right]=\left[c\right]\left[\varepsilon^{0}\right]$$

and

(3) $$\left[\varepsilon^{0}\right]={\left[c\right]}^{-1}\left[\sigma^{0}\right]$$

using Voigt’s notation, with $\left[\sigma^{0}\right]$ as the macroscopic stress vector and $\left[\varepsilon^{0}\right]$ as the macroscopic strain vector. For a general orthotropic material, the relationship

(4) $$\left[\begin{array}{c} \varepsilon_{xx}^{0}\\ \varepsilon_{yy}^{0}\\ \varepsilon_{zz}^{0}\\ \gamma_{yz}^{0}\\ \gamma_{xz}^{0}\\ \gamma_{xy}^{0} \end{array}\right]=\left[\begin{array}{cccccc} \frac{1}{E_{xx}} & \frac{-\nu_{yx}}{E_{yy}} & \frac{-\nu_{zx}}{E_{zz}} & 0 & 0 & 0\\ \; & \frac{1}{E_{yy}} & \frac{-\nu_{zy}}{E_{zz}} & 0 & 0 & 0\\ \; & \; & \frac{1}{E_{zz}} & 0 & 0 & 0\\ \; & \; & & \frac{1}{G_{yz}} & 0 & 0\\ \; & sym & \; & & \frac{1}{G_{xz}} & 0\\ \; & \; & & \; & & \frac{1}{G_{xy}} \end{array}\right]\left[\begin{array}{c} \sigma_{xx}^{0}\\ \sigma_{yy}^{0}\\ \sigma_{zz}^{0}\\ \sigma_{yz}^{0}\\ \sigma_{xz}^{0}\\ \sigma_{xy}^{0} \end{array}\right]$$

holds, with the elasticity matrix [c] being defined by nine independent material parameters. In addition to the three symmetry planes, the lattice cells exhibit an axial symmetry upon a 90° rotation about the axial direction z. This results in a reduction in the independent material parameters to only six. With $E_{yy}=E_{xx}$, $G_{yz}=G_{xz}$, and $\nu_{zy}=\nu_{zx}$, Equation (4) becomes

(5) $$\left[\begin{array}{c} \varepsilon_{xx}^{0}\\ \varepsilon_{yy}^{0}\\ \varepsilon_{zz}^{0}\\ \gamma_{yz}^{0}\\ \gamma_{xz}^{0}\\ \gamma_{xy}^{0} \end{array}\right]=\left[\begin{array}{cccccc} \frac{1}{E_{xx}} & \frac{-\nu_{yx}}{E_{xx}} & \frac{-\nu_{zx}}{E_{zz}} & 0 & 0 & 0\\ \; & \frac{1}{E_{xx}} & \frac{-\nu_{zx}}{E_{zz}} & 0 & 0 & 0\\ \; & \; & \frac{1}{E_{zz}} & 0 & 0 & 0\\ \; & \; & & \frac{1}{G_{xz}} & 0 & 0\\ \; & sym & \; & & \frac{1}{G_{xz}} & 0\\ \; & \; & & \; & & \frac{1}{G_{xy}} \end{array}\right]\left[\begin{array}{c} \sigma_{xx}^{0}\\ \sigma_{yy}^{0}\\ \sigma_{zz}^{0}\\ \sigma_{yz}^{0}\\ \sigma_{xz}^{0}\\ \sigma_{xy}^{0} \end{array}\right],$$

which represents the macroscopic material behavior of the orthotropic plate lattice structures with a 90° axisymmetry about the z-axis.

#### 3.1.1. Unit-Cell Models with Periodic Boundary Conditions

To determine the effective elastic material properties of the plate lattice metamaterials, representative volume elements (RVEs) in combination with periodic boundary conditions (PBCs) are used. As RVEs, the unit cells UC1, UC2, and UC3 depicted in Figure 1 are utilized.

The implementation of PBCs and the subsequent determination of effective structural properties are extensively described in [40]. For the application of PBCs to models with volume elements, the displacement degrees of freedom $u_{i}^{+}$ and $u_{i}^{-}$ of two opposing nodes are linked in the form

(6) $$u_{i}^{+}-u_{i}^{-}=\varepsilon_{ij}^{0}\Delta x_{j}.$$

The term $\varepsilon_{ij}^{0}$ in this context represents the macroscopic strain tensor, and $\Delta x_{j}$ denotes the distance between the two nodes. The components of the macroscopic strain tensor are introduced into the FEM model through additional dummy nodes. The degrees of freedom of these dummy nodes are associated with the macroscopic strains in such a way that each macroscopic strain component is described by a specific degree of freedom. Through this approach, Equation (6) can be formulated as a linear correlation among the degrees of freedom of three nodes. To apply a load, a value is specified at the corresponding degree of freedom of a dummy node that is connected to the desired macroscopic strain component. This leads to a reaction force occurring at this dummy node, which correlates with the applied macroscopic strain. As demonstrated in [40], macroscopic stresses can be determined from these reaction forces. The macroscopic transversal strains resulting from the load occur at the degrees of freedom of the dummy nodes at which no value is specified. The effective elastic material properties $E_{\mathrm{ii}}$ and $G_{\mathrm{ij}}$ can then be determined from the ratio of macroscopic stress to macroscopic strain. Poisson’s ratio $\nu_{\mathrm{ij}}$ can be calculated from the negative ratio of the effective transversal strain to the effective axial strain. To determine all six independent components of the effective elasticity matrix [c] of the lattice metamaterials, four independent linear elastic simulations are required:

- Uniaxial tensile loading in the x-direction;
- Uniaxial tensile loading in the z-direction;
- Pure shear loading in the xy-plane;
- Pure shear loading in the xz-plane.

**Figure 2:**
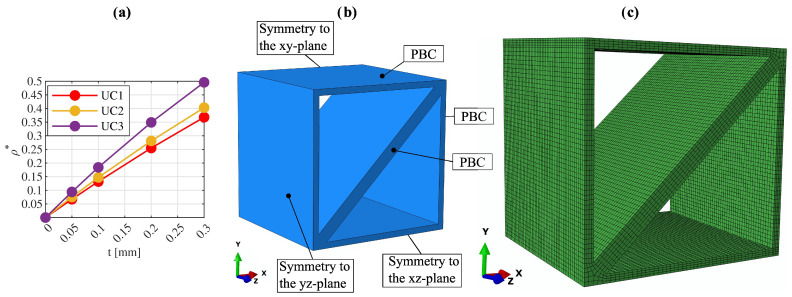
(**a**) Relative density $\rho^{*}\left(t\right)$, (**b**) geometric boundary conditions using three planes of symmetry, and (**c**) discretization of reduced model of unit cell UC1.

For the determination of the effective linear elastic material properties, it is irrelevant whether macroscopic strain or stress is acting as a load at the corresponding degrees of freedom of the dummy nodes. In the following, macroscopic stresses are applied and calculations are conducted for various unit cells with wall thicknesses of 0.05 mm, 0.1 mm, 0.2 mm, and 0.3 mm. Figure 2a shows the relationship between the wall thickness *t* and the corresponding relative density $\rho^{*}$.

To further enhance computational efficiency, the existing planes of symmetry of the unit cells are utilized in addition to periodicity, considering symmetries across the xy-, xz-, and yz-planes [38]. This allows the model size to be reduced to one-eighth of the unit cell. Figure 2b illustrates the geometric boundary conditions applied to the various sides of one-eighth of unit cell UC1.

Isoparametric linear hexahedral solid elements with an average element size of 0.05 mm are employed. A minimum of 4 elements are used through the wall thicknesses. A mesh convergence test showed that a further refinement of the discretization does not result in a significant change in the results. Particular attention is paid to minimizing distortion of the elements, especially in the areas of the plate intersections. Specifically, with the hexahedral elements used, no angles smaller than 50° or larger than 140° are used, and an aspect ratio below 5 is employed. Figure 2c shows the discretization of the reduced model of unit cell UC1. A comparison with solid elements with quadratic shape functions shows that the differences in the resulting effective stiffnesses are in the range of 1%.

In the case of unit cell UC3, it is possible to reduce the model to 1/64 of the original size. Unit cell UC3 consists of eight periodically repeating smaller unit cells with a volume of $V_{UC}=$ 2.5 mm × 2.5 mm × 2.5 mm. These minimal unit cells exhibit, again, three planes of symmetry.

#### 3.1.2. Modeling for the Characterization of the Anisotropy of the Lattice Metamaterials

If the stress–strain relationship from Equation (5) is known for a specific coordinate system, the associated complete fourth-order elasticity tensor $C_{ijkl}$ can be derived. This enables the generalized Hooke’s law to be represented in the tensor notation as

(7) $$\sigma_{ij}^{0}=C_{ijkl}\varepsilon_{kl}^{0},$$

which is an easier description for programming compared to Voigt’s notation. The quantities $\sigma_{ij}^{0}$ and $\varepsilon_{ij}^{0}$ represent the macroscopic stress tensor and the macroscopic strain tensor, respectively. The tensor $C_{ijkl}$ can be arbitrarily transformed using the rules of tensor transformation. This transformation is expressed by the equation

(8) $$C_{ijkl}^{*}=a_{ir}a_{js}a_{km}a_{ln}C_{rsmn},$$

where the terms $a_{ij}$ represent the transformation matrices. The components of the transformation matrices are the direction cosines

(9) $$a_{ij}=\cos\left(x_{i}^{*},x_{j}\right)$$

where $x_{i}^{*}$ and $x_{j}$ are the rotated and reference coordinate directions, respectively. This allows for determining the elastic behavior of the lattice cells under any arbitrary rotation of the reference system and, consequently, under any arbitrary loading direction. This is used to characterize the anisotropy of the lattice metamaterials by considering the anisotropy factor

(10) $$A=\frac{E_{max}}{E_{min}}.$$

#### 3.1.3. Unit-Cell Models of an Equilateral Hexagonal Honeycomb Metamaterial

For comparative purposes, the modeling and homogenization of a metamaterial consisting of honeycomb cells are undertaken. The considered honeycomb cell represents a regular hexagonal prism with side lengths and a height of 5 mm. To achieve comparable relative densities to those in the lattice cells, wall thickness variations are implemented at 0.4 mm, 0.6 mm, 0.8 mm, 1.2 mm, and 1.6 mm. The homogenization of the honeycomb cells is also performed with periodic boundary conditions [39,40] and isoparametric linear hexahedral solid elements.

#### 3.1.4. Modeling for the Optimization of the Wall Thicknesses

**Figure 3:**
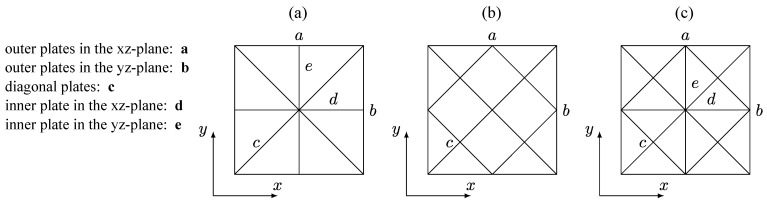
Naming of characteristic plates to be optimized for (**a**) unit cell UC1, (**b**) unit cell UC2, and (**c**) unit cell UC3.

The optimization of the wall thicknesses of the three unit cells is performed to enhance their weight-specific elastic properties. For this purpose, shell models with linear rectangular elements with a size of 0.1 mm are used, and a variation in wall thicknesses *t* between 0.1 mm and 0.3 mm is defined as an optimization condition. Figure 3 shows the division of the cells into areas, which should have the same wall thicknesses after optimization.

The optimization objective in this context is to achieve the stiffness optimization of the structures while maintaining constant weight. To fulfill this, the compliance of the considered structure is minimized, corresponding to a maximization of the stiffness. The compliance is determined through the strain energy $W_{el}$ of the entire considered structure [59,60], which is characterized in linear elasticity by

(11) $$W_{el}=\frac{1}{2}\int_{V_{S}}\sigma_{ij}\varepsilon_{ij}\;dV_{S}.$$

The cells are optimized separately with regard to uniaxial tensile loading in the x-direction, pure shear loading in the xy-plane, and pure shear loading in the xz-plane. Optimization with regard to uniaxial tensile loading in the z-direction is not necessary, as all plates are aligned in the z-direction, and the structure is already optimal for this load case.

Unit-cell models with periodic boundary conditions are used. This means that the outer plates of the cells have only half the wall thickness and a variation range of 0.05 mm to 0.15 mm. As the starting point of the parameter optimization, a constant wall thickness of t= 0.2 mm is used for all cells.

Due to targeted optimization with respect to a specific loading scenario, the 90° axisymmetry about the z-axis is no longer necessarily maintained after optimization. In this case, the optimized structure exhibits a general orthotropic material behavior.

Once the optimized wall thicknesses have been determined, a solid model with the new thicknesses is created, and the effective stiffnesses are determined similarly to the process in Section 3.1.1.

### 3.2. Models for Stability Analysis

Eigenvalue buckling analyses are carried out under a compressive load in the x-direction to characterize the stability behavior of the lattice metamaterials. For the discretization and approximation over a length of 5 mm, 50 isoparametric linear shell elements are utilized. Comparisons with quadratic shell elements have shown that the deviations of the resulting critical buckling loads are below 1%. A comparison with volume models has shown that the shell models yield a lower and thus more conservative critical buckling load. Two different unit-cell models and a multi-cell model are considered for the three lattice metamaterials.

#### 3.2.1. Unit-Cell Model with PBCs

An individual unit cell with periodic boundary conditions is under consideration to analyze the behavior of an infinitely large structure. This represents a metamaterial consisting of an infinite number of unit cells in each direction. Shell elements possess rotational degrees of freedom in addition to displacements. To apply PBCs, the rotational degrees of freedom $\phi_{i}^{+}$ and $\phi_{i}^{-}$ of two opposing nodes are coupled in the form

(12) $$\phi_{i}^{+}-\phi_{i}^{-}=0.$$

A macroscopic unit stress $\sigma_{xx}^{0}$ is applied as the loading condition. A characteristic of models with PBCs is that they suppress non-periodic buckling modes. This results from the fact that PBCs enforce equal deformations on opposite sides.

#### 3.2.2. Unit-Cell Model without PBCs

**Figure 4:**
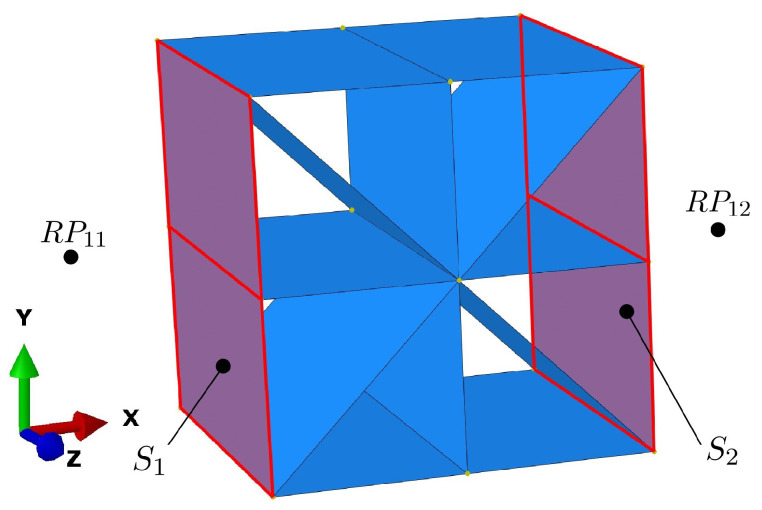
The coupling of faces $S_{1}$ and $S_{2}$ of the unit-cell model without PBCs to reference nodes.

A unit cell is modeled without PBC to investigate the buckling behavior of an isolated unit cell, which is not supported by other cells. In contrast to the models with PBCs, all walls of the cell are modeled with the full wall thickness of 0.2 mm. As shown in Figure 4, all nodes of the two opposite outer faces $S_{1}$ and $S_{2}$ of the unit cell perpendicular to the load direction are coupled with a reference point RP.

In particular, the translational degrees of freedom of the nodes of faces $S_{1}$ and $S_{2}$ are coupled to those of reference points $RP_{11}$ and $RP_{12}$, respectively. The rotational degrees of freedom are not coupled, allowing the plates oriented in the load direction to rotate. All translational degrees of freedom of $RP_{11}$ are set to zero, and the degrees of freedom $u_{y}$ and $u_{z}$ of $RP_{12}$ are set to zero. A unit pressure force in the x-direction is specified at the free degree of freedom of $RP_{12}$. This way, all nodes on side $S_{2}$ undergo the same displacement in the x-direction, as they are coupled to the corresponding displacement of the reference node. This very simple modeling approach corresponds to a cell that is clamped between two plates and is compressed.

#### 3.2.3. Multi-Cell Model without PBCs

In this modeling approach, a model consisting of 7 × 7 unit cells is constructed. The goal of this approach is to investigate the buckling behavior of a cell embedded in a larger structure and compare it with the results of modeling a unit cell with PBCs. For loading in the x-direction, a model is created with 7 unit cells in both the x- and y-directions. An expansion in the z-direction would only result in an elongation of the unit cells. Since this does not have as significant an impact on the local buckling of the unit cells as the expansion in the x- and y-directions, only a length of 5 mm is modeled in the z-direction. The same boundary conditions as those applied to the unit-cell model without PBCs are utilized using analogous reference points. For computational efficiency only 20 elements are used over a length of 5 mm as a mesh convergence test showed that a further refinement of the discretization does not result in a significant change of the critical buckling load.

### 3.3. Modeling for the Analysis of Stress Concentrations

To investigate the stress concentrations occurring in the lattice structures and the weight-specific strength, the detailed modeling of the cells with a wall thickness of 0.2 mm and radii instead of sharp notches at the plate intersections is conducted. Two different variants are considered. In the first variant, all plate intersections are modeled with a radius of R= 0.1 mm. In the second variant, for the edges where plates intersect at an angle of 90°, a radius of R= 0.2 mm is used instead of R= 0.1 mm. To assess the stress concentration issue, uniaxial tension in the x-direction is considered, and linear elastic calculations are carried out.

The reduced unit-cell models in Section 3.1.1, which were used for the determination of the effective stiffnesses, are utilized. Instead of linear elements, isoparametric hexahedral solid elements with quadratic shape functions and reduced integration with an average element size of 0.05 mm are employed to capture the stress concentrations accurately. A minimum of 4 elements are used through the wall thicknesses, and the radii are discretized with a minimum of 5 elements along the circumferential direction.

The uniaxial tensile loading is applied through a macroscopic stress specification at the respective dummy node, as described in Section 3.1.1.

## 4. Results and Discussion

### 4.1. The Homogenization of the Anisotropic Plate Lattice Structures

#### 4.1.1. Characterization of Anisotropic Elasticity

**Figure 5:**
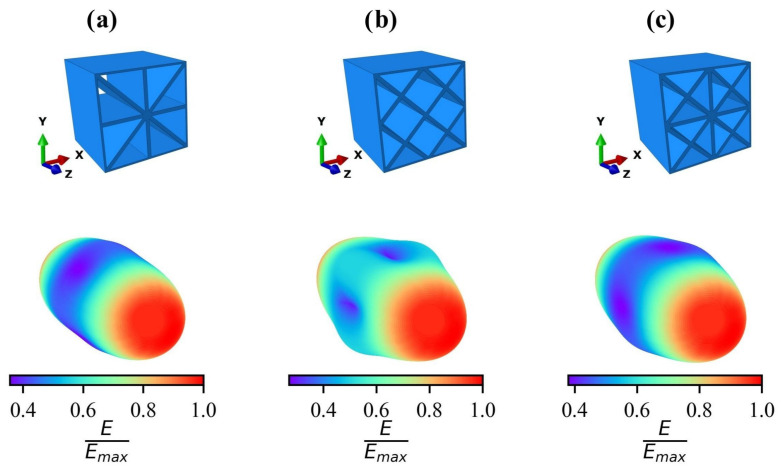
Lattice structures and the associated directionality of the normalized homogenized modulus of elasticity $E/E_{max}$ with the rotation of the coordinate system of the unit cells (**a**) UC1, (**b**) UC2, and (**c**) UC3 with a wall thickness of 0.2 mm.

Figure 5 shows the directionality of the effective homogenized stiffnesses $E(\theta_{1},\theta_{2})$ of the three unit cells UC1 (Figure 5a), UC2 (Figure 5b), and UC3 (Figure 5c) with a wall thickness of 0.2 mm, where $\theta_{1}$ and $\theta_{2}$ are spherical coordinates.

The color scale shown is normalized to the maximum occurring modulus of elasticity. It is clearly recognizable that all three unit cells show a maximum value of the modulus of elasticity in the z-direction. These maximum values coincide, with a deviation smaller than 1%, to the values determined through the Voigt bound [61], which was applied for cellular materials by [62] and provides the effective modulus of elasticity for the parallel connection of two isotropic linear elastic materials, which are, in the present case, AlSi10Mg and a gas. This confirms that the elastic material properties of the lattice structures can be accurately captured using volume elements. The dominance of the effective stiffness in the z-direction is due to the fact that all the plates of the cells are oriented in this direction. For cells UC2 and UC3, the minimum elastic modulus (dark purple areas in Figure 5a–c) occurs perpendicular to the z-axis in the x- or y-direction and, for cell UC1, at a rotation angle of 45° about the z-axis. This corresponds to a load on cell UC1 in the direction of the diagonal plates. Cell UC2 (Figure 5b) shows a local maximum in the direction with a rotation of 45° about the z-axis. This is due to the fact that all internal plates of cell UC2 are oriented in this direction. In contrast, a local maximum occurs in cell UC1 in the x- and y-directions, as the majority of the plates are oriented in these directions. In cell UC3, there is again a local maximum in the direction with a rotation of 45° about the z-axis, as the diagonal plates make a greater contribution to the stiffness than the horizontal or vertical plates.

Table 2 presents two different anisotropy factors for each of the three lattice structures. The factor $A_{max}$ describes the maximum existing anisotropy. The factor $A_{xy}$ characterizes the anisotropy present during a rotation around the z-axis in the xy-plane, providing a measure of how much the structure deviates from transverse isotropy. It is evident that cell UC2 exhibits the highest anisotropy factors, indicating the highest degree of anisotropy. Cells UC1 and UC3 display significantly less pronounced anisotropy. The analysis of $A_{xy}$ reveals that cells UC1 and UC3 exhibit material behavior similar to transverse isotropy, where $A_{xy}=$ 1.

#### 4.1.2. Effective Elastic Properties and Evaluation of Lightweight Potential

**Figure 6:**
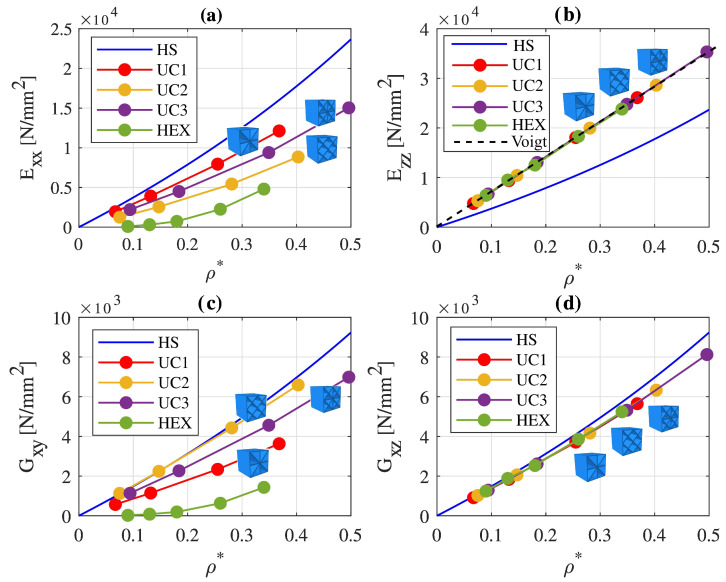
Effective elastic properties of the three lattice metamaterials in comparison with the effective properties of a honeycomb structure and the upper Hashin–Shtrikman bound: (**a**) Young’s modulus $E_{xx}$ in the x-direction, (**b**) Young’s modulus $E_{zz}$ in the z-direction, (**c**) shear modulus $G_{xy}$ in the xy-plane, (**d**) shear modulus $G_{xz}$ in the xz-plane.

Figure 6 illustrates the effective elastic material properties of the three lattice metamaterials, which result from homogenization with PBCs, as a function of the relative density.

Additionally depicted is the upper Hashin–Shtrikman bound, representing an upper limit for the maximally attainable elastic material constants of an isotropic cellular material [41], along with the effective material constants of a honeycomb cell. The points represent the specifically determined values for different wall thicknesses. Linear interpolation was applied between these points. In Figure 6a,b, the effective Young’s moduli $E_{xx}$ and $E_{zz}$ are presented as a function of the relative density. Figure 6c,d depict the shear moduli $G_{xy}$ and $G_{xz}$ as a function of the relative density. Due to the symmetry of unit cells UC1, UC2, and UC3, it holds that $E_{xx}=E_{yy}$ and $G_{xz}=G_{yz}$.

Figure 6a shows that unit cell UC1 has the highest weight-specific moduli of elasticity $E_{xx}$ compared to the other structures with the same relative density, whereas cell UC2 has the lowest values. This discrepancy arises from the load-case-dependent topology design of the cells. UC1 is tailored to exhibit superior stiffness properties under tensile/compressive loading in the x- or y-direction. In contrast, UC2 was chosen because of its optimized structure for shear loading, resulting in lower stiffness values under tensile loading. The additional inclined plates in UC3 compared to UC1 do not significantly enhance stiffness under tensile loading in the x-direction, as cells UC1 and UC3 have the same number of plates in the xy-plane. However, they contribute to increasing the weight. Consequently, the weight-specific modulus $E_{xx}$ of UC3 is lower than that of UC1.

For the elastic modulus $E_{zz}$ represented in Figure 6b, it is observed that UC1, UC2, and UC3 reach nearly identical values. The additional plates in UC2 and UC3 contribute to increased stiffness; however, this effect is counteracted by the additional material volume, resulting in more weight. This aligns with the Voigt bound [61], stating that the effective Young’s modulus of a composite of two materials connected in parallel depends solely on the elastic properties of the individual constituents and their volume fraction.

Figure 6c shows the weight-specific homogenized shear modulus $G_{xy}$. Cell UC2 attains the highest values, while UC1 exhibits the lowest. The shear modulus curve of UC3 lies between those of UC1 and UC2, as UC3 represents a superposition of the other UC1 and UC2.

Concerning the shear modulus $G_{xz}$ in Figure 6d, UC1, UC2, and UC3 possess almost identical weight-specific stiffnesses. It is noteworthy that UC2 exhibits similar values for shear moduli $G_{xy}$ and $G_{xz}$. The shear modulus $G_{xz}$ deviates by only approximately 4% to 10% from the shear modulus $G_{xy}$ for the considered wall thicknesses. This implies that the metamaterial consisting of cells UC2 exhibits similar stiffnesses under shear loading in the xy-plane, the xz-plane, and the yz-plane.

A comparison of the homogenized material properties of cells UC2 to UC3 with those of the honeycomb cell (green curves in Figure 6a–d) reveals that the properties of the honeycomb cell are approximately achieved or surpassed for all basic loading conditions. In particular, the Young’s moduli $E_{xx}$ and shear moduli $G_{xy}$ of the three unit cells are significantly higher than those of the honeycomb cell. The distributions of the elastic modulus $E_{xx}$ and the shear modulus $G_{xy}$ of the honeycomb cell exhibit a more pronounced nonlinear dependency on the relative density compared to the lattice structures. Consequently, with increasing relative density, the stiffness characteristics of the honeycomb cell increase more prominently than those of lattice metamaterials composed of cells UC1, UC2, and UC3.

The blue curves in Figure 6a–d represent the upper Hashin–Shtrikman bound (HS). The lattice structures presented in [19,25] reach this bound through a highly complex cell architecture. This complexity poses challenges for the additive manufacturing process, such as the removal of powder residues from closed cavities, significantly complicating the practical implementation of such lattice structures. From Figure 6a, it is evident that, while the elastic modulus $E_{xx}$ of the lattice cells is below the upper Hashin–Shtrikman bound, the elastic modulus $E_{zz}$ in Figure 6b significantly exceeds the upper Hashin–Shtrikman bound. This is possible due to the anisotropy of the considered structures. Figure 6c illustrates that the shear modulus $G_{xy}$ of unit cell UC2 is very close to the upper Hashin–Shtrikman bound. Examining the shear modulus $G_{xz}$ in Figure 6d shows that all three lattice cells reach a very similar value near the upper Hashin–Shtrikman bound. In particular, cell UC2 stands out, as it exhibits similar values close to the upper Hashin–Shtrikman bound for shear in the xy-, xz-, and yz-planes. The comparison of the homogenized properties of lattice structures with the upper Hashin–Shtrikman bound reveals that, through the deliberate exploitation of structural anisotropy, it is possible to use lattice structures with a topology that can be easily implemented in manufacturing, reaching or even exceeding the upper Hashin–Shtrikman bound for selectively chosen load cases.

**Figure 7:**
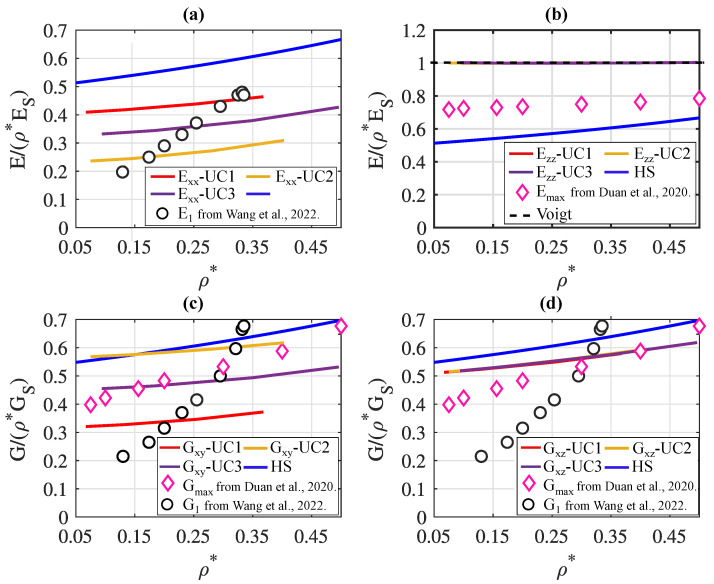
A comparison of the normalized (**a**) Young’s moduli $E_{xx}$, (**b**) Young’s moduli $E_{zz}$, (**c**) shear moduli $G_{xy}$, and (**d**) shear moduli $G_{xz}$ of cells UC1, UC2, and UC3 to the normalized effective stiffnesses of anisotropic plate lattice structures from Duan et al. [19] and Wang et al. [27].

In Figure 7, the effective stiffnesses of the three metamaterials consisting of cells UC1, UC2, and UC3, respectively, are compared with those of anisotropic plate lattice metamaterials from the literature [19,27], which were manufactured via the LPBF process. The stiffnesses are normalized with the elastic material constants $E_{S}$ and $G_{S}$ of the constituent material and the relative density $\rho^{*}$ to make them comparable, even though the various metamaterials are composed of different constituent materials. The structures discussed in [19,27] exhibit cubic symmetry. This implies that, for the stiffnesses of these structures, $E_{xx}=E_{yy}=E_{zz}$ and $G_{xy}=G_{xz}=G_{yz}$ hold, denoted hereafter by $E_{1}$ and $G_{1}$. Additionally, the upper Hashin–Shtrikman bound (HS) is plotted in all graphs in Figure 7.

Figure 7a displays the Young’s moduli $E_{xx}$ of lattice cells UC1, UC2, and UC3 and the stiffness $E_{1}$ of an anisotropic metamaterial from [27]. The depicted metamaterial from [27] has a constant wall thickness of 0.2 mm, and the variation in $\rho^{*}$ is achieved by changing the size of holes integrated into the structure. These holes are designed to facilitate de-powdering after manufacturing using the LPBF process. It is clearly visible that the curve of $E_{1}$ of the metamaterial from [27] exhibits a significant decrease with decreasing $\rho^{*}$, while the Young’s moduli $E_{xx}$ of structures UC1, UC2, and UC3 show a much flatter and thus more relative-density-independent trend. Below $\rho^{*}\approx 0.3$, the curve of UC1 is notably above that of the metamaterial from [27]. The value of $\rho^{*}=0.3$ corresponds to a wall thickness of cell UC1 between 0.2 mm and 0.3 mm. Additionally, the powder removal capability of the structures presented in [27] is dependent on the relative density, as it is largely determined by the size of the integrated holes. Thus, the highest stiffness values are only achievable at the expense of easy manufacturability.

In Figure 7b, the curves of the Young’s moduli $E_{zz}$ of cells UC1, UC2, and UC3 are depicted. According to Section 4.1.1, $E_{zz}$ represents the maximum Young’s modulus of these structures under a rotation of the reference system. In comparison to that, the maximum Young’s modulus $E_{max}$ of anisotropic plate lattice structures from [19] is presented. All structures discussed in [19] exhibit a similar architecture that can be transitioned into one another through the adjustment of two distinct geometric parameters. The plotted Young’s modulus $E_{max}$ represents the peak resulting from all parameter variations. The variation in relative density is achieved by adjusting the ratio of the wall thickness to the cell dimension, and de-powdering is facilitated by the introduction of additional small holes into the plates. It can be observed that both the curve of the structures from [19] and the curves of cells UC1, UC2, and UC3 are above the Hashin–Shtrikman bound, with the curves of cells UC1, UC2, and UC3 being at the Voigt bound, which is the maximum possible value.

Figure 7c compares the shear moduli $G_{xy}$ of cells UC1, UC2, and UC3 with the shear modulus $G_{1}$ of the structures from [27] and the maximum shear modulus $G_{max}$ of the structures from [19]. Similar to Young’s modulus $E_{1}$, a significant decrease in the shear modulus $G_{1}$ of structures from [27] is observed with decreasing $\rho^{*}$, while the curves of cells UC1, UC2, and UC3 exhibit a much more horizontal trend. Below $\rho^{*}\approx 0.325$, the shear modulus $G_{xy}$ of cell UC2 is higher than $G_{1}$. The value of $\rho^{*}=0.325$ corresponds to a wall thickness of cell UC2 between 0.2 mm and 0.3 mm. A comparison with the maximum shear modulus of the structures from [19] clearly indicates that the shear modulus $G_{xy}$ of cell UC2 reaches higher values over a wide range.

A similar trend is observed in Figure 7d, where the curves of the shear moduli $G_{xz}$ of cells UC1, UC2, and UC3 are plotted. In contrast to the shear modulus $G_{xy}$, all three cells exhibit approximately the same shear modulus $G_{xz}$, which extends over a wide range both above $G_{1}$ of the structures from [27] and above $G_{max}$ of the structures from [19].

#### 4.1.3. Optimization of Wall Thicknesses

**Figure 8:**
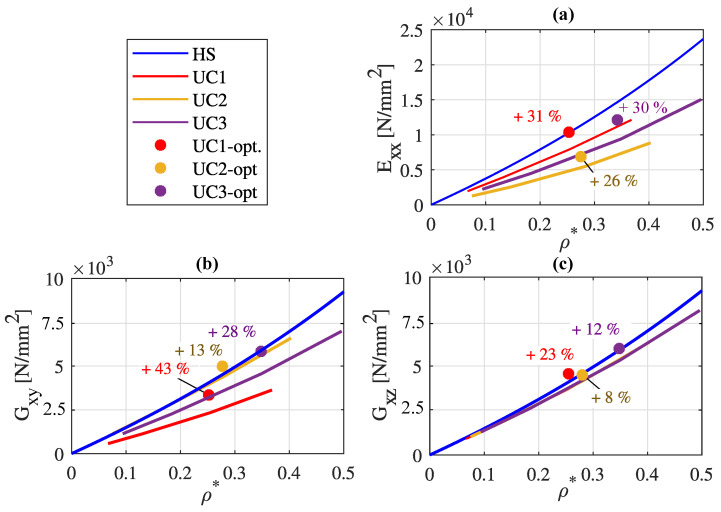
The effective elastic properties of the plate lattice metamaterials optimized for (**a**) pure tensile loading in the x-direction, (**b**) pure shear loading in the xy-plane, and (**c**) pure shear loading in the xz-plane and of the initial cells with a constant wall thickness of 0.2 mm compared to the upper Hashin–Shtrikman bound (HS).

An optimization of the wall thickness was carried out according to the objective function and constraints described in Section 3.1.4, originating from the base cell with a constant wall thickness of t= 0.2 mm. In Figure 8, the homogenized elastic material properties of the unit cells separately optimized with respect to individual primary load cases of tensile loading in the x-direction, pure shear loading in the xy-plane, and pure shear loading in the xz-plane are compared to the initial lattice properties of Figure 7 and the upper Hashin–Shtrikman bound (HS).

Figure 8a illustrates the optimized Young’s moduli $E_{xx}$ of the three unit cells, while Figure 8b and Figure 8c depict the optimized shear moduli $G_{xy}$ and $G_{xz}$. The individual optimized material properties are represented as points. Additionally, the percentage increases in the resulting homogenized material parameters are indicated.

Table 3 presents the corresponding optimized wall thicknesses of the characteristic plates of the three unit cells defined in Figure 3.

If the wall thicknesses of lattice regions *a* and *b*, or *d* and *e*, are not identical after optimization, the structure no longer exhibits a 90° axisymmetry about the z-axis. From Table 3 and Figure 3, it is apparent that the 90° axisymmetry about the z-axis is maintained in all three lattice structures after optimization for a shear loading in the xy-plane. Unit cell UC2 is the only cell where this symmetry is also preserved after optimization for tensile/compressive loading in the x-direction. After optimization with respect to a shear loading in the xz-plane, none of the cells exhibits a 90° axisymmetry about the z-axis anymore. Figure 8 shows that the targeted optimization of the unit cells with respect to a specific primary load results in a significant enhancement of material properties in all cases. The maximum achieved stiffness increase is 43%, observed in the case of cell UC2 optimized for pure shear loading in the xy-plane. It is obvious that the wall thicknesses specified in Table 3 are challenging to achieve with the LPBF process. Nevertheless, the diverse potential for improvement in mechanical properties through targeted optimization is evident. This underscores the importance of the continuous advancement of the additive manufacturing process.

### 4.2. The Stability Behavior of the Plate Lattice Structures under Compression Loading along the x-Direction

**Figure 9:**
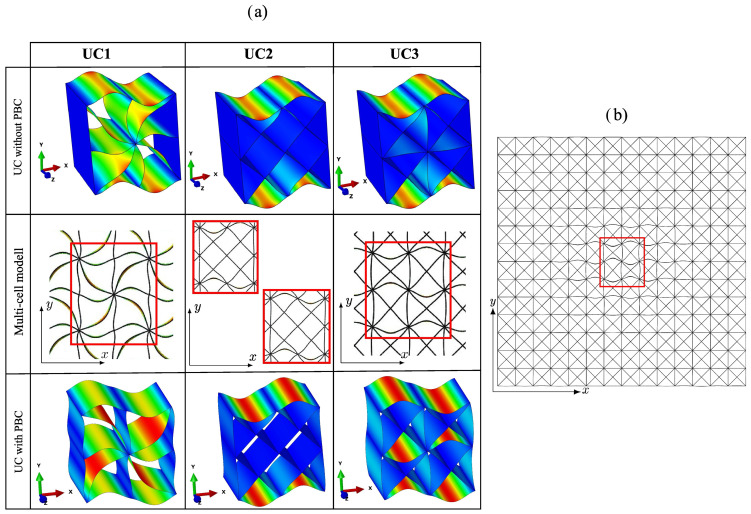
(**a**) A comparison of the analyzed buckling modes of the three plate lattice structures (t = 0.2 mm) under compression loading in the x-direction and various modeling approaches ($U=\sqrt{u_{x}^{2}+u_{y}^{2}+u_{z}^{2}}$ is depicted, with deformation increasing from blue to red) and (**b**) the entire multi-cell model of cell UC3.

In Figure 9a, buckling shapes resulting from an eigenvalue buckling analysis under compression loading in the x-direction are depicted for various model approaches. The corresponding eigenvalues are compared based on a predefined weight-specific stability criterion after the buckling shapes have been analyzed. In the case of unit-cell models, each represents the first buckling mode that emerges. For the multi-cell model, the buckling mode is considered in which the central cell buckles for the first time to eliminate edge effects. The central cell under consideration is highlighted in red.

Figure 9b illustrates the entire multi-cell model of cell UC3, with the central cell highlighted in red. Upon closer inspection of the central cell, it becomes apparent that the four quarters of the central cell, each representing a periodically extending structure itself, exhibit non-periodic buckling shapes due to the opposing sides deforming in opposite directions. In the case of unit-cell modeling with periodic boundary conditions (PBCs), if only one-quarter of the unit cell UC3 were used as an RVE, these non-periodic buckling modes would be suppressed by the PBCs. In contrast to this, the buckling shape shown in Figure 9b of the entire unit cell UC3 outlined in red is periodic and can also occur when PBCs are used.

In the multi-cell model of unit cell UC2, two buckling shapes occur with almost the same critical buckling load, as shown in Figure 9a in the second row and second column. The deviation is approx. 0.02%, which means that the different buckling modes have an almost equal probability of occurring. It is evident that one of the two possible buckling modes is a non-periodic buckling mode, which is in conflict with the PBCs. However, the second buckling mode occurring almost under the same load is periodic again.

As evident from Figure 9a, a similar fundamental buckling shape occurs for all three unit cells, regardless of the modeling variant. Hence, a very simple model, such as the unit-cell model without PBCs, can be sufficient to capture this fundamental buckling shape. Cell UC1 buckles in both the load-oriented and diagonal plates. In the case of cells UC2 and UC3, there is no buckling in the diagonal plates, as the effective buckling length of the diagonal plates is halved compared to cell UC1. The unit-cell models without PBCs exhibit slight differences compared to the multi-cell models and unit-cell models with PBCs. For the unit-cell model of cell UC3 with PBCs, the magnitude of the deformation of the middle and outer horizontal plates is almost equal, while for the unit-cell model without PBCs, the outer plates deform the most. Additionally, the models with PBCs show a slight deformation of the outer plates perpendicular to the load direction, which is not observed in the unit-cell models without PBCs due to the prescribed displacement boundary conditions. This deformation is also evident in the multi-cell models. In the models without PBCs, a slightly stronger deformation occurs at the edges in the z-direction of the buckling plates compared to the center of the plates. This is illustrated by the red areas at the edges of the buckling plates in the representations of the unit cells in the first row of Figure 9a. No such deformation enhancement at the plate edges is present in models with periodic boundary conditions.

A stability criterion is calculated to compare and assess the stability behavior of the three plate lattice structures. This criterion is based on a stability parameter,

(13) $$S_{YB}=\frac{\sigma_{Y}}{\sigma_{b}^{el}},$$

which is the ratio of the yield stress $\sigma_{Y}$ to the elastic stress $\sigma_{b}^{el}$. The stress $\sigma_{b}^{el}$ is the stress that occurs in a linear elastic FEM calculation in the plates, in which buckling happens when the critical buckling load is applied. The von Mises equivalent stress is considered for $\sigma_{b}^{el}$. The yield-before-buckling criterion is defined by

(14) $$S_{YB}<1.$$

This critical stability limit represents the threshold at which buckling precedes yielding and is not reached as long as $\sigma_{Y}<\sigma_{b}^{el}$ holds. A similar approach is presented in [19].

**Figure 10:**
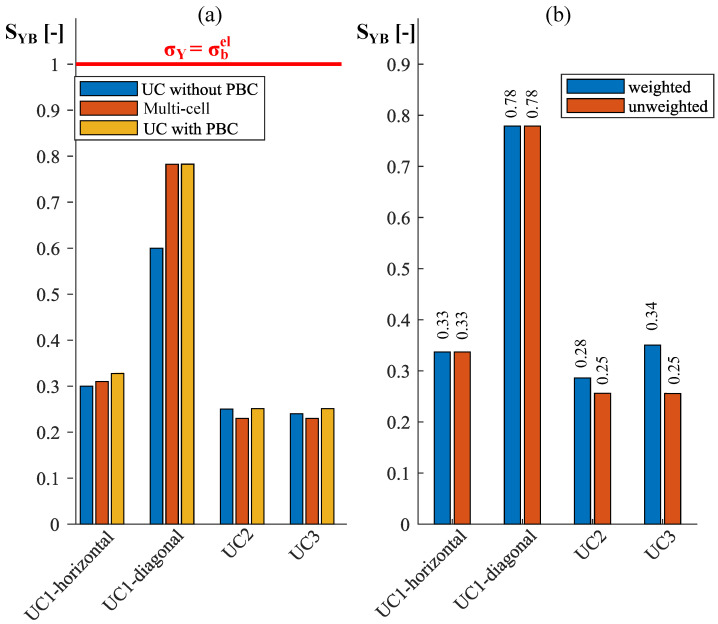
(**a**) Stability parameters $S_{YB}$ of the three lattice unit cells (t = 0.2 mm) under compression loading in the x-direction, depending on the different modeling approaches, and (**b**) a comparison of the unweighted and weight-specific stability parameters obtained by unit-cell models with PBCs.

Figure 10a depicts the stability parameters obtained for the three unit cells UC1, UC2, and UC3 with a wall thickness of 0.2 mm depending on the different modeling variants. From Figure 9a, it is apparent that both the diagonal and horizontal plates buckle in cell UC1. Therefore, two distinct parameters are determined by calculating $\sigma_{b}^{el}$ separately for the horizontal and diagonal plates. While the horizontal plates exhibit largely constant stress in the linear elastic simulation for all unit cells, this stress varies in the diagonal plates of cell UC1 due to bending, both along the plate’s longitudinal direction and across its thickness. Consequently, the stress $\sigma_{b}^{el}$ in the diagonal plates is determined as the average of the magnitudes of the stresses prevailing on the tension and compression sides in the middle of the diagonal plates as a conservative approach. In unit cells UC2 and UC3, only the horizontal plates exhibit buckling. Figure 10a shows that cell UC1 is the most susceptible to buckling, with the diagonal plates standing out in particular. The stability parameter of the diagonal plates of cell UC1 is, depending on the model variant, between approx. two and three times as high as that of unit cells UC2 and UC3.

This means that unit cell UC1 is two to three times more susceptible to buckling. Nevertheless, all calculated stability parameters are below the critical limit of 1, which is shown as a red line in Figure 10a. Furthermore, it can be seen that, for all cells, all three model variants provide very similar stability parameters. This fact, along with the knowledge derived from Figure 9 that the utilization of PBCs with unit cells UC1, UC2, and UC3 as RVEs does not suppress non-periodic buckling modes, allows the consideration of a unit-cell model with PBCs instead of a complex multi-cell model. This facilitates a substantial reduction in the effort required to model a cell representing an RVE of a larger structure.

Since, during the previous stability considerations, all unit cells had the same wall thickness, the material volume $V_{S}$ and, due to the constant density of the base material, the weight continue to increase from cell UC1 up to cell UC3. To account for this in the context of a weight-specific comparison of the stability parameters of the three lattice structures, the determined parameters are multiplied by a weighting factor. The weighting factor used is the multiplier listed in Table 4, representing the ratio between the material volumes $V_{S}$ of the three unit cells. Weighting with the relative density $\rho^{*}$ would yield the same relations.

Figure 10b compares the weighted and unweighted stability parameters obtained by using the unit-cell models of UC1, UC2, and UC3 with PBCs. While the stability characteristics of unit cell UC1 remain constant, the characteristics of cells UC2 and UC3 undergo changes. Because of the higher weighting factor of unit cell UC3, its stability parameter experiences a more pronounced alteration compared to that of unit cell UC2. Consequently, although the unweighted values of cells UC2 and UC3 are nearly identical across all considered buckling scenarios, a more distinct difference emerges after weighting, as depicted in Figure 10b. Post-weighting, the stability parameters of unit cell UC2 are notably lower. This implies that cell UC2 exhibits superior weight-specific stability properties compared to the other two cells.

Stability analyses were also carried out for a compressive load in the z-direction and for a shear load in the xy-plane. These results are listed in the Appendix A.

### 4.3. Influence of Different Notch Radii on the Stress Concentrations and Effective Stiffnesses

**Figure 11:**
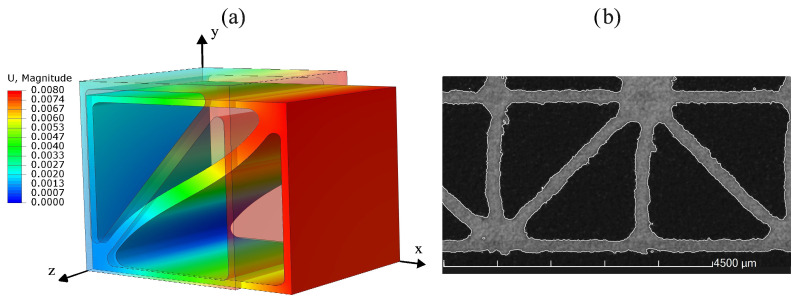
(**a**) The displacement $U=\sqrt{u_{x}^{2}+u_{y}^{2}+u_{z}^{2}}$ in mm of the reduced model of unit cell UC1 modeled with PBCs and symmetry constraints under tensile loading in the x-direction and (**b**) a CT scan of a manufactured specimen composed of unit cells of type UC1 with radii at the intersections.

Figure 11a shows the deformation of the reduced model of unit cell UC1 resulting from tensile loading in the x-direction, taking into account the periodicity and the three symmetry planes. The undeformed cell is shown in transparent color. The elongation of the cell in the x-direction resulting from the tensile loading and the corresponding transversal contraction in the y- and z-directions is visible. Figure 11b shows a CT scan of a specimen composed of unit cells of type UC1 with a wall thickness of 0.2 mm and with radii of R= 0.1 mm and R= 0.2 mm at the 45° and 90° intersections, respectively.

**Figure 12:**
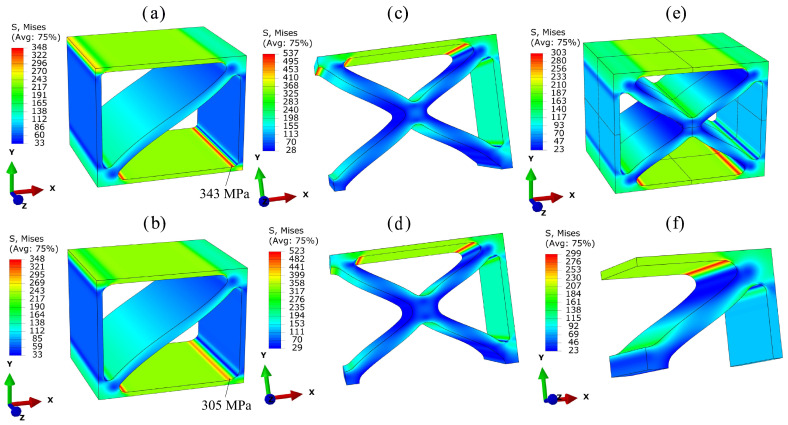
Deformed structures and von Mises equivalent stresses (in MPa) of (**a**,**b**) unit cell UC1, (**c**,**d**) unit cell UC2, and (**e**,**f**) unit cell UC3 under the same uniaxial macroscopic tensile load of 26 MPa in the x-direction, where the models in the first row each have a radius of R = 0.1 mm at all intersections, and the models in the second row deviate from this by having a radius of R = 0.2 mm at all 90° intersections.

Figure 12 shows the deformed structures and the von Mises equivalent stresses of the three unit cells resulting from a uniaxial tensile load in the x-direction.

The upper row presents the resulting stresses when modeling the cells with a radius of R= 0.1 mm at all intersections. In the models in the lower row, intersections at which plates meet at an angle of 90° are modeled with a radius of R= 0.2 mm and the remaining intersections with R= 0.1 mm. For cells UC1 (Figure 12a,b) and UC2 (Figure 12c,d), one-eighth of the unit cell is modeled considering the reflectional symmetries. In the case of cell UC3, Figure 12e also depicts an eighth of the original cell, but Figure 12f shows the minimum model of cell UC3 using all periodicities and reflectional symmetries. The same macroscopic tensile stress $\sigma_{xx}^{0}=$ 26 MPa was applied to all cells. This corresponds to a force in the x-direction of 650 N acting on the model of the entire unit cell at the associated dummy node.

Cell UC1, which was chosen for tensile loading in the x-direction, exhibits significantly lower stresses both in the notches and in the x-oriented plates compared to cell UC2, which is not intended for tensile loading. Cell UC3 shows similar stress values to those of cell UC1.

**Figure 13:**
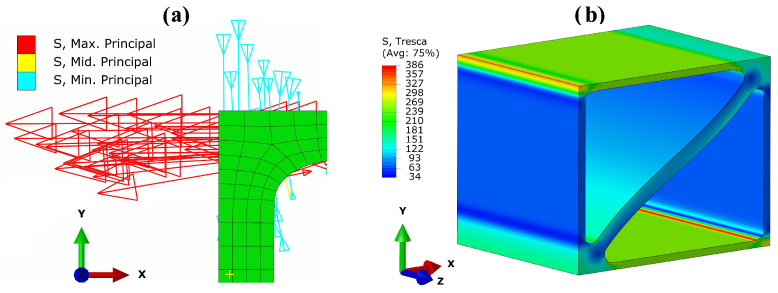
(**a**) Principal stress directions and (**b**) Tresca equivalent stress $\sigma_{T}=2\tau_{max}$ of cell UC1 with a radius of R = 0.1 mm at all intersections under a macroscopic tensile load of 26 MPa in the x-direction.

The comparison between Figure 12a and Figure 12b for unit cell UC1 shows an 11% reduction in notch stresses, decreasing from 343 MPa to 305 MPa, if the larger radius is used. When using the smaller radius at the 90° intersections, stress concentrations occur in the center of these intersections (orange areas at the upper-left and lower-right edges of the model in Figure 12a). These stress concentrations are primarily caused by shear stresses and represent a special case, as they occur within the solid material rather than at the surface. Figure 13 illustrates the formation of these stress concentrations. In Figure 13a, the principal stresses and their directions resulting from the macroscopic tensile load in the x-direction are shown as an arrow plot in a detailed view of one of the affected intersections of cell UC1. The minimum principal stress $\sigma_{3}$ (S, Min. Principal) represented by turquoise arrows is present as compressive stress in the y-direction in the plate oriented in the yz-plane, while it assumes a value of approx. zero in the plate oriented in the xz-plane. In contrast, the maximum principal stress $\sigma_{1}$ (S, Max. Principal), represented by red arrows, is present as tensile stress in the x-direction in the plate oriented in the xz-plane and becomes approx. zero in the plate oriented in the yz-plane. Both stresses $\sigma_{1}$ and $\sigma_{3}$ are present at the intersection. These two principal stresses result in the maximum principal shear stress,

(15) $$\tau_{max}=\frac{|\sigma_{1}-\sigma_{3}|}{2},$$

which acts in a plane perpendicular to the bisector of the x- and y-axes. In Figure 13b, the Tresca equivalent stress $\sigma_{T}=2\tau_{max}$ resulting from uniaxial tensile loading in the x-direction is depicted. The concentration of the shear stress $\tau_{max}$ at the center of the 90° intersections is clearly visible at the upper-left edge of the model in Figure 13b. The stress concentrations resulting from $\tau_{max}$ within the von Mises equivalent stress can be completely eliminated, as shown in Figure 12b, by using a radius of R = 0.2 mm at the 90° intersections.

Another reduction in notch stresses at intersections where plates meet at an angle less than 90° could be achieved by using a radius of R = 0.2 mm there as well. However, this would lead to increased localized material accumulations, which increase the weight and complicate the manufacturing process. For this reason, this is omitted for all three lattice cells, and a radius of R= 0.1 mm is suggested for these 45° intersections.

The results of cell UC2 are shown in Figure 12c,d. They reveal that the notch stresses prominently visible in the upper-left radius of the model in Figure 12c can be completely eliminated by using a radius of R=0.2 mm at this edge instead of R=0.1 mm. Since neither Figure 12c nor Figure 12d shows stress concentrations at the central intersection in the middle of the depicted models, the use of the larger radius can be omitted in that region, resulting in weight savings.

As observed in the case of cell UC2, the models of cell UC3 displayed in Figure 12e,f demonstrate that there is no stress concentration at the central intersection, whether a radius of R=0.1 mm or R=0.2 mm is utilized. Since this central intersection is the only location in cell UC3 where plates meet at a 90° angle, and thus the only location where a radius of 0.2 mm is considered, the use of the larger radius can be entirely omitted for cell UC3.

The introduction of the radii changes the material volume $V_{S}$ and thus also the weight of the cells compared to the previously considered cell configuration without radii. The percentage increase in material volume $\Delta V_{S}$ compared to the reference cell without radii is shown in Table 5.

The absolute values of the effective Young’s modulus $E_{xx}$ and the normalized Young’s modulus $E_{xx}^{*}=E_{xx}\;/\;\rho^{*}$ are also depicted. The first column contains values determined based on the reference configuration without radii. The values in the second and third columns correspond to models with a constant radius of 0.1 mm and models with a combination of radii of 0.1 mm and 0.2 mm, respectively. The incorporation of radii is found to increase the weight but concurrently enhances Young’s modulus. An examination of $E_{xx}^{*}$ reveals that the integration of radii improves the ratio of Young’s modulus to weight, as they introduce an additional stiffening effect in the regions of plate intersections. Thus, the inclusion of radii provides added value in terms of achievable strength and weight-specific stiffness.

For the comparison of the weight-specific strengths of the three lattice metamaterials, the factor

(16) $$\Phi =\frac{1}{\sigma_{vM}^{max}\rho^{*}}\;\cdot \;100$$

is defined. The term $\sigma_{vM}^{max}$ represents the maximum local von Mises equivalent stress occurring in each of the three lattice cells under the application of the same macroscopic stress $\sigma_{xx}^{0}$. The term $\rho^{*}$ is the relative density of the unit cell altered due to the radius. The larger the value of Φ, the lower the weighted stress $\sigma_{vM}^{max}\rho^{*}$ resulting from the loading, and the higher the expected specific strength of the lattice structure. The factor 100 is merely a multiplicative constant in order to obtain easily representable values. The resulting values are depicted in Table 6 depending on the selected radius *R*. It can be observed that cell UC1 exhibits the highest specific strength, while cell UC2 has the lowest. The chosen radii have little influence on the specific strength. This is due to the fact that the maximum stress occurs at an intersection point in all three cells, where the same radius is maintained in both variants. This results in only minimal changes to the maximum stress, which are largely compensated for by weighting with the relative density $\rho^{*}$.

For a more precise comparison of stress concentrations, stress concentration factors

(17) $$K_{t}=\frac{\sigma_{vM}^{local}}{\sigma_{vM}^{0}}=\frac{\sigma_{vM}^{local}}{\sigma_{xx}^{0}}$$

are determined for the three cells. These factors represent the ratio of a resulting local von Mises equivalent stress $\sigma_{vM}^{local}$ to the applied macroscopic von Mises equivalent stress $\sigma_{vM}^{0}$, where, in the case of uniaxial tension, $\sigma_{vM}^{0}=\sigma_{xx}^{0}$. The factor $K_{t}$ describes the stress amplification relative to the effective stress $\sigma_{xx}^{0}$, which arises from both the lattice structure and the notch geometry.

For all three cells, the factor $K_{t}^{max}$ is determined, where $\sigma_{vM}^{local}=\sigma_{vM}^{max}$. In the case of cell UC1, an additional factor $K_{t}^{90{}^{\circ}-intersection}$ is evaluated. This factor uses the maximum stress present at the center of the 90° intersections in cell UC1 (orange areas at the upper-left and lower-right edges of the model in Figure 12a) as $\sigma_{vM}^{local}$. Additionally, the factor $K_{t}^{radius}$ is calculated for cells UC1 and UC2. In the case of cell UC1, this factor uses the maximum stress at the radii of the 90° intersections (red area in the lower-right radius of the model in Figure 12a) as $\sigma_{vM}^{local}$. For cell UC2, the maximum stress present at the radii of the outer 90° intersections (red area in the upper-left radius of the model in Figure 12c) is utilized. As there is no change in the notch stresses for cell UC3 due to the altered radii, only the factor $K_{t}^{max}$ is calculated. All of these factors are presented in Table 6.

For all three cells, the change in radii has only a minor influence on the global stress concentration factor $K_{t}^{max}$, with cell UC2 exhibiting the highest value. The value of the stress concentration factor $K_{t}^{max}$ of cell UC2 is approx. 1.5 and approx. 1.8 times greater than those of cells UC1 and UC3, respectively. The different notch radii do have a significant influence on the stress concentration factors $K_{t}^{90{}^{\circ}-intersection}$ and $K_{t}^{radius}$. For cell UC1, both factors can be reduced by using the larger radius. For cell UC2, the use of the larger radius allows for a reduction in the factor $K_{t}^{radius}$ by 30%.

## 5. Conclusions

Three different half-open anisotropic 2.5D plate lattice metamaterials with a straightforward architecture for ease of additive manufacturing with the LPBF technique were analyzed. These three investigated metamaterials are characterized by their different unit cells. These three cells were labeled as UC1, UC2, and UC3 and exhibit an orthotropic material behavior with a 90° axisymmetry about the axial direction z. All cells are intended to be additively manufactured using the LPBF process with the aluminum alloy AlSi10Mg. A wall thickness ranging between 0.1 mm and 0.3 mm is targeted for lightweight design purposes. In addition to the investigation of lattice cells with a uniform wall thickness, an optimization of the wall thicknesses was carried out for further improvement of the lightweight properties.

A comparison of the effective elastic material properties of the anisotropic plate lattice metamaterials with those of a honeycomb metamaterial reveals that all three lattice materials achieve or surpass the honeycomb structure, even with uniform wall thicknesses. A comparison with the upper Hashin–Shtrikman bound shows that, with the deliberate utilization of material anisotropy with a relatively simple architecture with a uniform wall thickness and without closed cavities, it is possible to reach and even exceed the upper Hashin–Shtrikman bound for certain loading directions. Other anisotropic plate lattice structures discussed in the literature [19,27] can be surpassed not only in terms of ease of manufacturability but also concerning their weight-specific effective stiffnesses under specific loads. These comparisons, combined with simple de-powdering, demonstrate the high lightweight potential and practical relevance of the analyzed 2.5D plate lattice metamaterials.

The optimization of the wall thicknesses leads to a significant increase in weight-specific effective elastic material properties. In the case of optimizing cell UC1 for shear loading in the xy-plane, an increase in weight-specific shear stiffness of up to 43% is achievable.

The investigation of the stability behavior of the plate lattice cells with a constant wall thickness using a linear buckling analysis reveals the following:

- It is shown that, by selecting appropriate representative volume elements, no buckling modes are suppressed by using periodic boundary conditions.
- In all plate lattice structures, plastic deformation occurs prior to stability failure under compressive loading both in the axial direction and perpendicular to it, as well as under shear loading in the plane perpendicular to the axial direction.
- Unit cell UC2 exhibits the highest weight-specific stability parameters for buckling under compressive loading in the axial direction or perpendicular to it, as well as for buckling under shear loading in the plane perpendicular to the axial direction.

The analysis of stress concentrations in the plate lattice structures indicates that the introduction of radii at the plate intersections can reduce the occurring stress peaks and simultaneously increase the weight-specific stiffnesses. By using a radius of R= 0.2 mm instead of R= 0.1 mm, the stress concentration factors can be reduced by up to 30% in the case of cell UC2. Furthermore, the utilization of a radius of 0.2 mm effectively prevents stress concentrations from occurring at the centers of 90° intersections of unit cell UC1.

In the future, specimens of the developed lattice structures will be fabricated using the Laser Powder Bed Fusion (LPBF) process, and experimental testing will be used to investigate how much the numerical results deviate from the weight-specific mechanical properties of manufactured specimens. 

## Figures and Tables

**Table 1:** The material properties of AlSi10Mg used in the simulations [11,51,52].

*E* [N ${\mathrm{mm}}^{-2}$]	ν [-]	$\sigma_{Y}$ [N ${\mathrm{mm}}^{-2}$]
71,000	0.34	272

**Table 2:** The anisotropy factors of the plate lattice cells (t= 0.2 mm).

	UC1	UC2	UC3
$A_{max}$ [-]	2.8	3.7	2.6
$A_{xy}$ [-]	1.2	1.9	1.2

**Table 3:** Wall thicknesses *t* of the unit cells after optimization with respect to the load cases of tensile loading in the x-direction, pure shear loading in the xy-plane, and pure shear loading in the xz-plane.

	*t* [mm] for Tensile Loading in x-Direction			*t* [mm] for Shear Loading in xy-Plane			*t* [mm] for Shear Loading in xz-Plane		
Lattice Regions of Figure 3	UC1	UC2	UC3	UC1	UC2	UC3	UC1	UC2	UC3
*a*	0.150	0.150	0.150	0.065	0.050	0.050	0.150	0.150	0.150
*b*	0.065	0.150	0.100	0.065	0.050	0.050	0.050	0.050	0.050
*c*	0.180	0.160	0.160	0.300	0.240	0.270	0.200	0.200	0.200
*d*	0.300	-	0.300	0.130	-	0.100	0.300	-	0.300
*e*	0.130	-	0.200	0.130	-	0.100	0.100	-	0.100

**Table 4:** Material volumes $V_{S}$ and corresponding weighting factors of the three unit cells with a wall thickness of 0.2 mm.

	UC1	UC2	UC3
Volume [$mm^{3}$]	31.81	35.15	43.62
Weighting factor [-]	1.000	1.105	1.371

**Table 5:** Volume and effective Young’s modulus $E_{xx}$ depending on the selected radius *R*.

		Reference	R= 0.1 mm	R= 0.1 mm/0.2 mm
UC1	$\Delta V_{S}$ [%]	0	3	4
	$E_{xx}$ [MPa]	7920	8449	8562
	$E_{xx}^{*}$ [MPa]	31,122	32,130	32,292
UC2	$\Delta V_{S}$ [%]	0	3	6
	$E_{xx}$ [MPa]	5413	5975	6111
	$E_{xx}^{*}$ [MPa]	19,141	20,434	20,441
UC3	$\Delta V_{S}$ [%]	0	5	6
	$E_{xx}$ [MPa]	9371	10,487	10,587
	$E_{xx}^{*}$ [MPa]	26,978	28,773	28,713

**Table 6:** Weight-specific strength factor Φ and stress concentration factors $K_{t}$ depending on the selected radius *R*.

		R= 0.1 mm	R= 0.1 mm/0.2 mm
UC1	Φ [$mm^{2}N^{-1}$]	1.09	1.08
	$K_{t}^{max}$ [-]	13.38	13.38
	$K_{t}^{90{}^{\circ}-intersection}$ [-]	10.77	9.12
	$K_{t}^{radius}$ [-]	13.19	11.73
UC2	Φ [$mm^{2}N^{-1}$]	0.64	0.64
	$K_{t}^{max}$ [-]	20.65	20.11
	$K_{t}^{radius}$ [-]	20.38	14.23
UC3	Φ [$mm^{2}N^{-1}$]	0.91	0.91
	$K_{t}^{max}$ [-]	11.65	11.50

**Figure A1:**
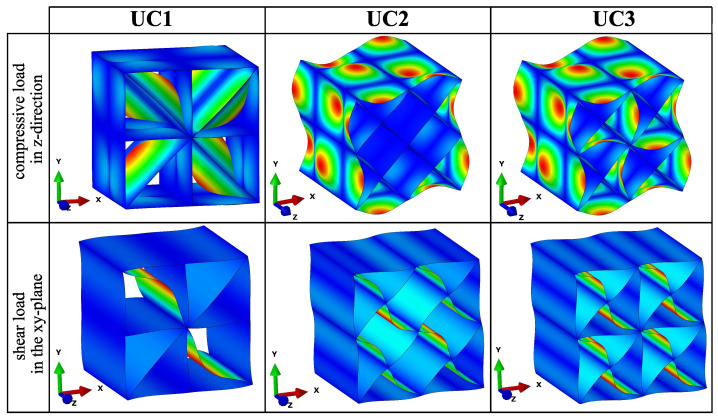
The first buckling modes resulting from a linear buckling analysis of the three lattice structures (t = 0.2 mm) obtained by unit-cell models with PBCs under a uniaxial compressive load in the z-direction and a pure shear load in the xy-plane ($U=\sqrt{u_{x}^{2}+u_{y}^{2}+u_{z}^{2}}$ are depicted, with deformation increasing from blue to red).

**Figure A2:**
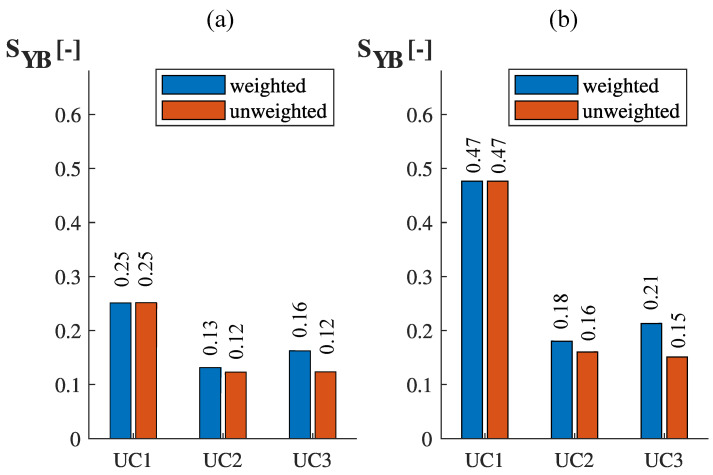
Unweighted and weight-specific stability parameters $S_{YB}$ of the three lattice structures (t = 0.2 mm) obtained by unit-cell models with PBCs for (**a**) uniaxial compressive loading in the z-direction and (**b**) pure shear loading in the xy-plane.

## Data Availability

Data are contained within the article.

## Appendix A

Figure A1 shows the buckling modes of the three unit cells with a wall thickness of t= 0.2 mm under a uniaxial compressive load in the x-direction and under a pure shear load in the xy-plane. The shapes shown were determined using unit-cell models with periodic boundary conditions (PBCs).

Figure A2 shows the corresponding weighted and unweighted stability parameters $S_{YB}$ of the three lattice structures. Figure A2a depicts the stability parameters for buckling under a compressive load in the z-direction, and Figure A2b represents the parameters for buckling under a shear load in the xy-plane.
